# Secondary contact seeds phenotypic novelty in cichlid fishes

**DOI:** 10.1098/rspb.2014.2272

**Published:** 2015-01-07

**Authors:** Paul Nichols, Martin J. Genner, Cock van Oosterhout, Alan Smith, Paul Parsons, Harold Sungani, Jennifer Swanstrom, Domino A. Joyce

**Affiliations:** 1 School of Biological, Biomedical and Environmental Sciences, University of Hull, Hull HU6 7RX, UK; 2 School of Biological Sciences, Life Sciences Building, 24 Tyndall Avenue, Bristol BS8 1TQ, UK; 3 School of Environmental Science, Norwich Research Park, University of East Anglia, Norwich NR4 7TJ, UK

**Keywords:** introgression, admixture, secondary contact, phenotypic novelty, haplochromine fishes, river capture

## Abstract

Theory proposes that genomic admixture between formerly reproductively isolated populations can generate phenotypic novelty for selection to act upon. Secondary contact may therefore be a significant promoter of phenotypic novelty that allows species to overcome environmental challenges and adapt to novel environments, including during adaptive radiation. To date, this has largely been considered from the perspective of interspecific hybridization at contact zones. However, it is also possible that this process occurs more commonly between natural populations of a single species, and thus its importance in adaptive evolution may have been underestimated. In this study, we tested the consequences of genomic introgression during apparent secondary contact between phenotypically similar lineages of the riverine cichlid fish *Astatotilapia calliptera*. We provide population genetic evidence of a secondary contact zone in the wild, and then demonstrate using mate-choice experiments that both lineages can reproduce together successfully in laboratory conditions. Finally, we show that genomically admixed individuals display extreme phenotypes not observed in the parental lineages. Collectively, the evidence shows that secondary contact can drive the evolution of phenotypic novelty, suggesting that pulses of secondary contact may repeatedly seed genetic novelty, which when coupled with ecological opportunity could promote rapid adaptive evolution in natural circumstances.

## 1. Introduction

Genomic introgression is an important evolutionary process that can generate variation in behaviour, life-history traits and morphology [1–5]. It can do so more rapidly than mutation because, unlike mutation, introgression involves the exchange of genetic material that has already been tested against one of the parental genomic backgrounds. Introgression can produce phenotypes that are more extreme than either parent, a concept known as transgressive segregation [6]. In first-generation offspring, recessive deleterious mutations fixed in the parental species can be masked, resulting in heterosis or hybrid vigour. Although this effect dissipates with the loss in heterozygosity in subsequent generations, the novel gene combinations in the transgressive segregants will remain, and they may provide a novel substrate for natural and sexual selection. There is evidence that transgressive segregation arises from complementary gene action of additive alleles with opposing effect that are present in multiple loci in the parental species [6]. When recombination brings together alleles of similar effect at different loci, this will result in an extreme phenotype [6–8]. Such transgressive phenotypes tend to be reported as the offspring of occasional events between completely reproductively isolated species. However, the same principles could apply to cases of secondary contact among populations previously geographically separated that have undergone divergent adaptive evolution of ecologically significant phenotypic traits. If so, then the importance of transgressive segregation in enabling populations to rapidly adapt to environmental change could have been widely overlooked.

Haplochromine cichlid flocks are widely distributed across sub-Saharan Africa, and the communities found in the Great Lakes of Malawi and Victoria have long been textbook examples of vertebrate adaptive radiation. Interspecific hybridization has been reported within these radiations using molecular markers [9–11], and interspecific hybrids are known to exhibit phenotypic novelty distinct from parental species, in both body and jaw morphology [12,13]. Here, we use the term phenotypic novelty in a similar manner to Mayr [14] to refer to ‘acquired structures or properties that could permit the performance of a new function’. Thus, it is possible that introgression among formerly allopatric lineages of the same species could also give rise to phenotypic novelty and that, ultimately, this could facilitate the invasion of new niches or adaptive zones.

**Figure 1.**
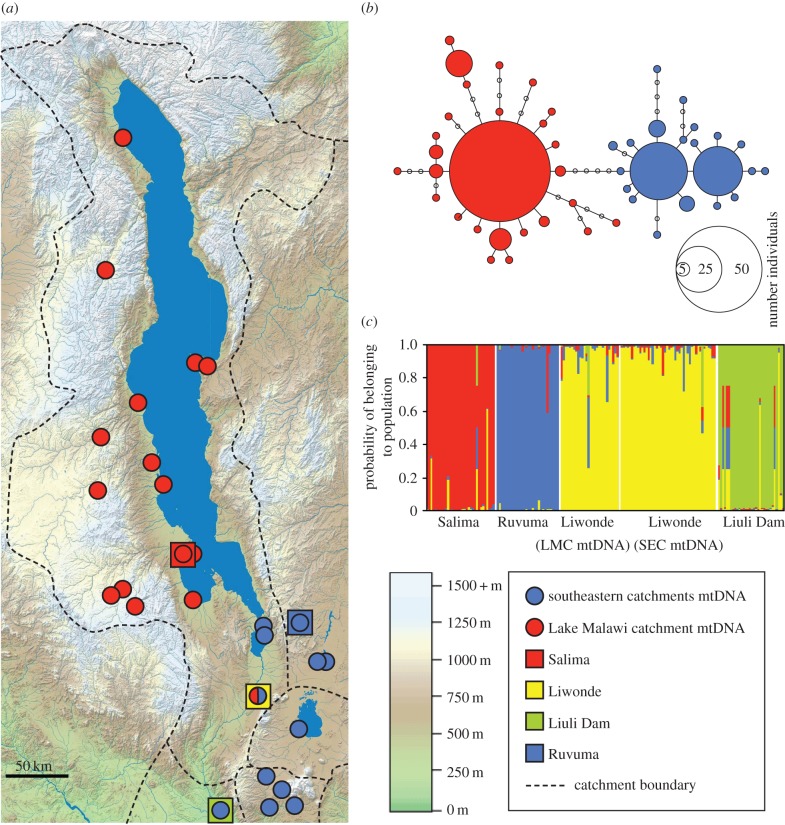
Spatial population genetic structure of *A. calliptera* in the Lake Malawi region. (*a*,*b*) Each of the two mitochondrial haplogroups are spatially restricted, to either the Lake Malawi catchment (LMC; red circles) or southeastern catchments (SEC; blue circles). The haplogroups are found in contact only at Liwonde on Upper Shire River. Squares indicate populations screened at microsatellite markers. (*c*) Populations sampled from four locations were identified as strongly genetically distinct using microsatellite loci, with the two mtDNA lineages freely interbreeding at the Liwonde contact zone. (Online version in colour.)

We previously identified two mitochondrial DNA lineages of the generalist riverine cichlid *Astatotilapia calliptera* [11], which is one of only two riverine representatives of the Lake Malawi haplochromine flock not endemic to the lake itself. In this study, we show that the distributions of these mitochondrial lineages are largely allopatric, except in the south of the Lake Malawi catchment (LMC) where they have achieved secondary contact (figure 1). We exploited this as a system to investigate whether the populations are reproductively isolated, and whether novel intraspecific transgressive phenotypes could evolve through reproduction on secondary contact. We show that when this contact is reconstructed in laboratory conditions, new transgressive phenotypes arise suggesting a wider role for secondary contact in the evolution of new species traits. Our results are consistent with the hypothesis that intraspecific secondary contact can produce novel phenotypes, and the prevalence of transgressive segregation maybe underestimated in nature, potentially enabling species to overcome new environmental changes through rapid introgression-driven adaptive evolution.

## 2. Material and methods

### (a) Study species

*Astatotilapia calliptera* has a wide distribution encompassing the LMC, Lake Chilwa, and the eastward flowing Lower Zambezi, Pungwe, Buzi, lower Save and Ruvuma systems [15]. The species exhibits allopatric variation in breeding male coloration and morphology, and it has been cautiously noted that allopatric colour varients may represent distinct species [11], although no studies fully supported this hypothesis. The only mate-choice trials among allopatric forms to date have shown partial assortative mating among two of three tested populations [16]. Thus, we consider these allopatric populations of *A. calliptera* differing in colour and ecomorphological traits to be intraspecific variants.

### (b) Phylogeography

Twenty-five *A. calliptera* populations were sampled across the Lake Malawi, Ruvuma, Lake Chilwa, Ruo and Lower Shire catchments (mean sample size 8, range 1–23; figure 1*a*, electronic supplementary material, table S1). New samples for this study (204 individuals from 24 populations) were collected as fin clips and preserved in 100% ethanol. Promega DNA ‘Wizard’ kits were used to extract DNA and partial sequence of the mtDNA D-loop was amplified and sequenced following published methods [17]. The sequences were aligned using ClustalW in Dambe [18], and the alignment of the 210 individuals comprised 403 base pairs and 117 haplotypes. These new sequences have GenBank accession numbers (KJ742942–KJ743145). Using Dambe, the sites with gaps or unresolved bases were removed, leaving the final alignment used for analysis comprising 357 base pairs and 48 haplotypes. A mitochondrial control region haplotype network was reconstructed using Hapstar v. 2.2 [19] with a distance matrix generated in Arlequin v. 2.1 [20].

Two distinct mtDNA haplogroups were present in the dataset, which could be separated with a diagnostic restriction enzyme (HPAI). This was used to screen an additional 400 individuals collected from Liwonde to separate them into their respective haplogroups. Samples of both haplogroups from Liwonde, together with samples from three other sites (electronic supplementary material, tables S1 and S2, and figure S1) were genotyped at seven microsatellite loci UNH154 [21] Pzeb3, Pzeb5 [22], TMOM5, TMOM11 [23] Ppun5, Ppun7 and PPUN21 [24]. Microsatellite alleles were sized using Tandem [25] and checked for scoring error and null alleles using Microchecker [26].

Structure [27] was used to explore population genetic structure, employing 500 000 Markov chain Monte Carlo generations with a burn-in of 50 000 generations. The most likely value of *K* (number of populations) was identified as 4 using the online program Structure Harvester [28], after testing *K* = 1–12 with each test repeated 10 times. Population pairwise *F*_ST_ was calculated with Genepop [29] (electronic supplementary material, table S3). Ima v. 2 [30] was used to estimate migration rates between each pair of populations [31] combining both the nuclear and mitochondrial DNA datasets. We completed two runs for each population pair with 80 chains and high heating to promote mixing between adjacent pairs of chains.

### (c) Mate-choice experiments

Pre-mating behavioural barriers between putative parental *A. calliptera* lineages were examined using behavioural female mate-choice assays. Experiments were carried out on pure bred laboratory stock of *A. calliptera* ‘Ruvuma’ and ‘Salima’ collected from the field in 2005 and 2007, respectively, and then maintained at the University of Hull in large stock tanks within a recirculation system. Prior to the experiment, ‘Ruvuma’ and ‘Salima’ lineages had not been in contact. Fish were fed once a day on dry food. Temperature was maintained between 24°C and 27°C. The light regime was one of 12 L : 12 D using full spectrum fluorescent tubes.

A partial partition design was used with a 6 m long tank (6 × 0.8 × 0.35 m) split into eight compartments of equal size. Walls between the compartments were made from plastic mesh with apertures large enough to permit female fish to pass between the compartments yet small enough to restrict males' access to them. Prior to being introduced to the experiment, all fish were tagged using Passive Integral Transponder tags so individual fish could be identified. Procedures were carried out one month before experiments were due to begin and in accordance with Home Office protocol (UK Home Office project licence number—PPL60/4036). In each replicate, three compartments contained a single male *A. calliptera* ‘Ruvuma’ and three contained a single male *A. calliptera* from ‘Salima’. Two compartments behaved as refuges for female fish.

Some cichlids exhibit multiple paternity [20]. If this is the case in *A. calliptera*, a second mating decision in the experiment may be skewed by those males in close proximity. Male position in the tank compartments was therefore determined using a pseudo-random-block design, ensuring males from the same lineage were not assigned to adjacent compartments. Six female fish from each *A. calliptera* lineage were placed in the tank and could exercise free choice of males. Four replicates were carried out in series between March and June 2010. Fish were not re-used. After a minimum of eight of the twelve female fish present in the experiment spawned, the mouthbrooding females were stripped of their eggs and paternity was established by screening a minimum of eight developing fry per brood at five of the microsatellite loci (Pzeb5, UNH154, TMOM5, TMOM11 and Ppun21). DNA was extracted using the ‘hotshot’ method [32] from the fin tissue of the 24 adult fish and from 288 developing eggs; brood sizes range from 24 to 80 (electronic supplementary material, table S4).

### (d) Common garden experiments and morphology

To quantify phenotypic variation in hybrid lines relative to that of parental lineages, reference purebred lines, hybrids and backcrosses were produced and reared under standard conditions. First-generation (F_1_) purebred and hybrid fish were reared from wild-caught parental stock. These were then crossed using a Punnett square design (electronic supplementary material, table S5) to generate F_2_ offspring with all potential parental combinations. Most crosses were duplicated, and crosses were obtained ‘each way’ with respect to lineage of the male participant. Broods were split to account for tank effects and fish were grown under standard conditions as follows. Each aquarium (10 × 30 × 10 cm) contained 10 fish of a particular cross and were fed once a day with: (i) sinking pellets of size 0.5 mm for the first three weeks, and then (ii) flake food and sinking pellets of size 1.0 mm over alternating days. All broods were reared within the same re-circulating aquarium system with 12 L : 12 D light regime and water temperature between 24°C and 27°C. Fish were reared until they measured 35 mm (±5 mm) before being sacrificed using an immersion overdose of anaesthetic in accordance with UK Home Office procedure. To assess morphological variation among individuals, we used landmark-based morphometrics. Digital images were captured using an 18MP Canon EOS550D camera mounted on a static camera rig. Images were taken of the left lateral side with fish in standard, left-facing orientation with the mouth closed [33–35]. Pins were placed behind the first and last rays of the dorsal fin, first ray of the anal fin and the first ray of the pelvic fin. Digital images were uploaded to tpsDig [36] and 25 landmarks were marked (electronic supplementary material, figure S2). Geometric morphometric analyses were performed using standard procedure and default settings in tpsRelw [37] (electronic supplementary material, table S6). Initially, coordinate data generated using tpsDig were subjected to a Procrustes alignment, before a partial warp analysis, and finally a relative warp analysis which generated relative warp scores for analysis. Deformation grids created in tpsRelw were used to illustrate the variation captured along the major relative warp axes. All relative warp score axes showed significant associations with body size (centroid size), however this explained less than 10% of observed variation on all axes (electronic supplementary material, table S7). To correct for this effect, in all subsequent analyses we used residuals of the relationship between relative warp scores and centroid size. Transgressive segregation was defined as the extent of phenotypic novelty in hybrid crosses relative to the total parental range along each relative warp axis. To account for unequal sample size between parental and hybrid lines, which would artificially affect the phenotypic space range, a bootstrapping approach was used to test for statistically higher variation in hybrids relative to parental lines. Specifically, we tested for significant extensions of phenotypic space range in each group of hybrid offspring by comparing the phenotypic range occupied in the sample of parental fish (*n* = 50) to the range occupied by each of 500 random bootstrap samples of 50 individuals.

## 3. Results

### (a) Phylogeography

Forty-eight mtDNA control region haplotypes in two clearly divergent haplogroups were found within the 210 individuals of *A. calliptera* sequenced (figure 1*b*). One haplogroup was found exclusively in the LMC, while the second was present in the river systems of the southeastern catchments (SEC), namely the Ruvuma, Lake Chilwa, Ruo and Lower Shire. The two haplogroups were found in sympatry at Liwonde near the south of the LMC. The four *A. calliptera* populations screened at nuclear microsatellite loci were significantly genetically different (*F*_ST_ range 0.0524–0.2394; all comparisons *p* < 0.001; electronic supplementary material, tables S2 and S3). Individuals at Liwonde possessing LMC mtDNA were not genetically different from those with SEC mtDNA (*F*_ST_ = 0.0001; *p* = 0.53). Structure did not differentiate individuals from the two haplogroups at this contact point (figure 1*c*). Coalescent analyses of the directionality of gene flow together with the spatial distribution of the haplogroups suggest that the Liwonde contact zone has been colonized from both the LMC and SEC regions (figure 1*a*; electronic supplementary material, figure S1).

### (b) Mate-choice experiments

**Figure 2.**
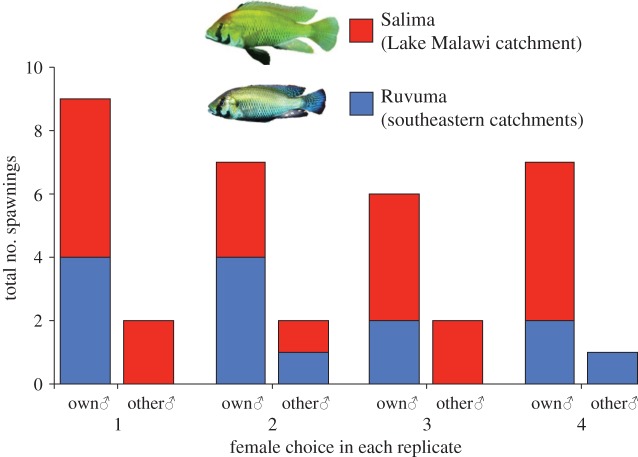
Mate choice of *A. calliptera*. In each of the four experimental replicates where females were given a free choice of males, they showed an overall but not complete preference for males from their own population. (Online version in colour.)

Reproductively receptive females significantly preferred to mate with males from their own population in each of four replicate trials (binomial tests: replicate 1, *p* = 0.0059; replicate 2, *p* = 0.0195; replicate 3, *p* = 0.0351; replicate 4, *p* < 0.0001; figure 2). On average, 19.5% (range 12.5–25%) of matings were with males from the other population.

### (c) Common garden experiments and morphology

**Figure 3.**
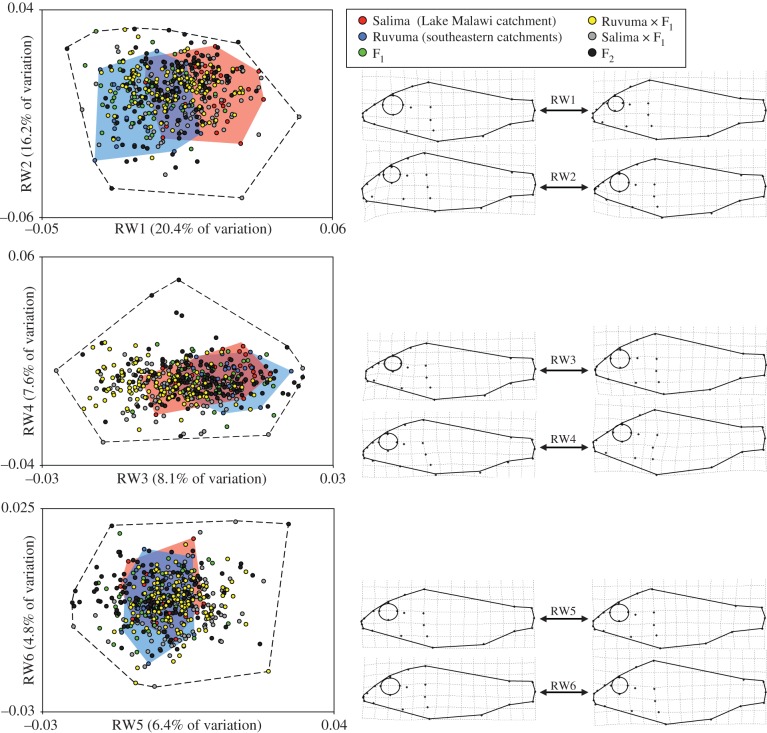
Size-corrected morphological space occupied by laboratory-reared pure bred and hybrid *A. calliptera*. The percentage of variation captured by each relative warp axis is indicated on the axis label. The dashed line encompasses the total morphological space occupied by all individuals. Deformation grids illustrate the extreme morphological variation observed. (Online version in colour.)

In total, the first six axes captured 63.5% of the total observed variation. The range of morphological variation observed in hybrid lineages was greater than observed in parental populations on all axes (figure 3). Significant transgressive segregation was observed along four of the first six relative warp axes (table 1). Morphological change associated with relative warps captured changes to eye size, snout length and body depth.

**Table 1.** Extent of transgression observed in hybrids along six primary axes of body-size corrected morphospace. (Transgression is reported as the median percentage increase in axis space of hybrid lines relative to parental lines (Salima and Ruvuma pooled) within 500 bootstrap replicates (*n* = 50 individuals). Statistical significance of transgression is derived from the proportion of replicates where transgression was observed. *p < 0.05, **p < 0.01, ***p < 0.001.)

relative warp axis	hybrid cross			
	F_1_	Salima × F_1_	Ruvuma × F_1_	F_2_
RW1	5.7	7.6	0	0
RW2	13.8*	17.1	8.8	21.3**
RW3	12.0**	37.8***	34.4***	34.6***
RW4	20.2*	27.8**	13.3	84.8*
RW5	33.9***	61.4***	43.2***	92.0***
RW6	4.6	14.4	6.2	10.8

## 4. Discussion

Parental lineages studied here showed considerable overlap in morphology, and only partially mated assortatively so are likely to be allopatric variants of the same species, *A. calliptera*. Thus, the results highlight how secondary contact of formerly allopatric populations can lead to phenotypic novelty. The novelty reported here could have adaptive significance in wild populations, given that it affects ecologically relevant traits in African cichlids. Specifically, morphological characters that showed evidence of transgressive segregation, including body depth, head shape and eye size have been shown to be correlated with ecological niche use in cichlids [38,39]. The data support the assertion that diversification observed in laboratory conditions could be ecologically relevant and determine individual fitness in the natural environment.

Our experiments show that intraspecific morphological variation among *A. calliptera* populations is at least partially genetically based, despite considerable overlap between populations. Given this evidence, we hypothesize that the allopatric ancestral riverine phenotypes have been under strong stabilizing selection and that over time, positive and negative mutations have accumulated in linkage blocks because they cancelled out phenotypic effects. We suggest that when genetic exchange occurred between lineages, it acted to breakdown these geographically localized linkage blocks, releasing the epistatic variance into additive variation and allowing positive and negative mutations to segregate freely, in turn resulting in extreme F_2_ phenotypes. We examined collections of wild individuals from Salima, the Ruvuma headwaters and Liwonde, to look for evidence of transgressive head and body shape morphology in wild populations (electronic supplementary material, tables S8 and S9). Our results did provide some evidence of a greater phenotype range in the Liwonde population relative to combined Ruvuma and Salima populations in the first four relative warp axes (electronic supplementary material, figure S3 and table S10), consistent with the Liwonde population having greater phenotypic variance. However, cichlid eco-phenotypes in the natural environment are mediated by local environmental conditions and developmental plasticity in morphological characters is prevalent in cichlids [40] so we are extremely cautious in interpreting this as evidence of wild transgressive phenotypes. Local phenotypes are likely to have been under strong stabilizing or directional selection since any initial hybridization events.

We studied gene flow across the boundary between the LMC and adjacent river systems. Traditionally, such catchment boundaries around African lakes have been presumed to be relatively impermeable, leading to suggestions that lake faunas are monophyletic [41]. The results of this study, alongside other recent discoveries [11], enable us to question this assumption. It is likely that periods of genetic connectivity have taken place across catchment boundaries during river capture events or periods of flooding [9,42]. We are uncertain when the last period of connectivity between our study populations in Lake Malawi and the Ruvuma river would have been, but evidence that the Shire River and Lake Malombe (now maximum 7 m depth) were dry between 1500 AD and 1850 AD [43,44], together with the geographically restricted nature of the eastern haplogroup in the Lake Malawi basin, suggests these colonizations of the LMC may have taken place within the last 200 years. There is increasing evidence that periodic hybridization among genetically divergent riverine haplochromines has been a feature of African cichlid evolution [9] and this could be an effective means of transferring standing genetic variation across geographical areas and potentially among species [42]. Selection of ecologically adaptive genetic material from standing genetic variation after secondary contact has been termed the ‘transporter hypothesis' and may explain the relative speed with which ecological speciation can sometimes occur [45]. Individuals may benefit from genetic components with a selective advantage already tested in a parental genetic background, and do not need to await the accumulation of beneficial mutations.

The importance of interspecific hybridization in the evolution of phenotypic novelty has been increasingly recognized. For example, hybridization among species of *Heliconius* butterflies has been identified as a means of exchanging advantageous mimicry pattern genomic regions among species [1]. Interspecific hybridization has also been suggested to be a trigger for adaptive radiation, and there is phylogenetic evidence consistent with this process in several cases of adaptive evolution, including cichlid fishes [10]. Notably, experimental laboratory crosses using cichlids have shown that the extent of morphological diversity observed in hybrid crosses correlates positively with the extent of genetic divergence of the parental lineages [35] and that the extent of divergence observed between hybrids of a radiation can even predict the total morphological diversity within radiations [46]. Although the importance of interspecific hybridization for providing phenotypic novelty is becoming increasing recognized, the ability for admixture events to promote the evolution of new phenotypes between populations of the same species has received comparably less attention. An exception to this has been the consideration of intraspecific hybridization and invasive species [47]. There is strong evidence from plants that multiple introductions facilitate invasions [48] and this is thought to be through the positive effects of various processes including new gene interactions and the transfer of favourable genes. For example, multiple introductions have been shown to be key for the evolutionary potential of a highly invasive snail, *Melanoides tuburculata* [49]. Our study suggests that transgressive segregation following periods of population segregation may also occur within the natural range of species. Typically, such contact zones between lineages that were formerly geographically separated are identified and studied once reproductive isolation can be detected. However, populations that have diverged without reproductive isolation are likely to be both more common, and more difficult to detect (e.g. [50]). In such circumstances, injections of allopatric variation could generate pulses of new recombination blocks which provide heritable variation and new potential for adaptive change.

In this study, we have shown that periodic genetic leaking of the Lake Malawi boundaries allows cryptic intraspecific hybridization of formerly allopatric lineages, and can produce the substrate upon which natural and sexual selection could potentially act. This changes our current understanding of the potential of hybridization to generate biodiversity by suggesting that in any biological system, temporally separated waves of invasion/secondary contact could result in genomic admixture which ultimately seeds novel phenotypic diversity and adaptive change in response to environmental change.

## Supplementary Material

Secondary contact seeds phenotypic novelty in cichlid fishes - additional information and data

## Data accessibility

Sequences have been deposited in GenBank under accession numbers (KJ742942–KJ743145), other data are available in the electronic supplementary material.
