# Supplementary material for: Secondary contact seeds phenotypic novelty in cichlid fishes
Source: Proc Biol Sci. 2015 Jan 7;282(1798):20142272. doi: 10.1098/rspb.2014.2272 (PMC4262179; doi:10.1098/rspb.2014.2272)
Supplement: Secondary contact seeds phenotypic novelty in cichlid fishes - additional information and data [file rspb20142272supp1.docx]

**Electronic Supplementary Material:**

Nichols *et al.* Secondary contact seeds phenotypic novelty in cichlid fishes

**Table S1. Sampling localities and numbers of individuals sequenced for mitochondrial DNA**

|  | Latitude | Longitude | Catchment | N | Sampling Date | Collector  Reference | Accession  numbers | Haplotype numbers from network alignment (no. found where >1) |
| --- | --- | --- | --- | --- | --- | --- | --- | --- |
|  |  |  |  |  |  |  |  |  |
| Lake Chilwa | 15° 22' 19'' S | 35° 35' 27'' E | Lake Chilwa | 11 | Jul-05 | PN, MG | KJ742955-65 | 4(7), 5, 6(2), 7. |
| Chisumulu Island | 12° 1' 36'' S | 34° 37' 45'' E | Lake Malawi | 4 | Jul-06 | PN | KJ742966-70 | 1, 8, 9, 2(2) |
| Dwangwa River, Kasungu | 13° 3' 48'' S | 33° 28' 58'' E | Lake Malawi | 18 | Sep-06 | MG | KJ742971-88 | 10(10), 11(5), 12, 13, 14 |
| Kamuzu Reservoir | 14° 10' 26'' S | 33° 38' 35'' E | Lake Malawi | 12 | Apr-04 | MG, GFT | KJ743050-61 | 2 (11), 22 |
| Linthipe | 14° 8' 16'' S | 34° 5' 17'' E | Lake Malawi | 5 | Apr-04 | MG, GFT | KJ743077-87 | 2(4), 32 |
| South Rukuru, Enukweni | 11° 11' 16'' S | 33° 52' 54'' E | Lake Malawi | 13 | Apr-04 | MG, GFT | KJ742989-43001 | 15(13) |
| Lake Chilingali | 12° 55' 19'' S | 34° 12' 43'' E | Lake Malawi | 12 | Jul-04 | MG PN | KJ743002-13 | 2(6), 16(2), 17, 18(3) |
| Chia Lagoon | 13° 7' 5'' S | 34° 19' 4'' E | Lake Malawi | 4 | Oct-04 | MG, GFT | KJ742951-54 | 2(4) |
| Salima* | 13° 46' 9'' S | 34° 26' 8'' E | Lake Malawi | 13 | Apr-05 | MG | KJ743126-38 | 2(12), 47 |
| Likoma Island | 12°3' 12" S | 34° 45' 08" E | Lake Malawi | 11 | Jul-06 | PN | KJ743066-76 | 1(2), 2(2), 26(2), 28, 29, 30, 31 |
| Bua River | 13° 19' 33'' S | 33° 26' 17'' E | Lake Malawi | 9 | Sep-06 | MG | KJ742942-50 | 1(2), 2(6), 3 |
| Kambiri Point | 13° 46' 43'' S | 34° 37' 20'' E | Lake Malawi | 3 | May-09 | MG, JS, AS | KJ743047, KJ743049 | 2(3) |
| Mtakataka | 14° 12' 33'' S | 34° 30' 47'' E | Lake Malawi | 1 | May-09 | MG, JS, AS | KJ743045 | 2 |
| Unaka Lagoon | 12° 23' 30'' S | 34° 5' 19'' E | Lake Malawi | 7 | Jul-04, Jul-06 | MG, PN | KJ743139-45 | 16, 48 |
| Karonga | 9° 56' 8.73'' S | 33° 56' 47'' E | Lake Malawi | 6 | Unknown | Shaw *et al.* (2000) | AY911722-23, AF298938-41 | 2(3), 45, 46(2) |
| Liuli Dam* | 16° 2' 16'' S | 34° 50' 27'' E | Lower Shire | 6 | Jul-05 | MG, PN | KJ743082-87 | 19(6) |
| Mangochi Hills (Kwiputi)* | 14° 22' 27'' S | 35° 32' 55'' E | Ruvuma | 19 | Oct-04, May-09 | PN, MG, JS, AS | KJ743021-3, KJ743026-7, KJ743029, KJ743031, KJ743062-125 | 6(12), 19(2), 23, 24, 25, 44(2) |
| Lake Chiuta | 14° 43' 44" S | 35° 51' 39" E | Ruvuma | 2 | May-09 | MG, JS, AS | KJ743024, KJ743030 | 6(2) |
| Nkhowe (Lake Chiuta) | 14° 42' 40'' S | 35° 51' 10'' E | Ruvuma | 1 | May-09 | MG, JS, AS | KJ743028 | 6 |
| Kwakwasi River | 16° 2' 7'' S | 35° 15' 12'' E | Ruo | 4 | May-09 | MG, JS, AS | KJ743034, KJ743037, KJ743039, KJ743041 | 6, 20(3) |
| Likabula headwaters | 16° 0' 58'' S | 35° 29' 30'' E | Ruo | 1 | May-09 | MG, JS, AS | KJ743042 | 6 |
| Luchenza River | 15° 50' 46'' S | 35° 11' 36'' E | Ruo | 3 | May-09 | MG, JS, AS | KJ743035, KJ743038, KJ743040 | 6(3) |
| Thuchila River | 15° 55' 40'' S | 35° 21' 49'' E | Ruo | 3 | May-09 | MG, JS, AS | KJ743032-3, KJ743043 | 20(3) |
| Liwonde* | 15° 3' 13'' S | 35° 13' 10'' E | Upper Shire | 25 | Jul-05, May-09 | MG, PN, JS, AS | KJ743014, KJ743018, KJ743019, KJ743036, KJ743088-43101. | 6(6), 19(12), 21, 33, 34, 35, 36, 37, 38 |
| Mangochi | 14° 40' 53'' S | 35° 11' 30'' E | Upper Shire | 23 | Jul-05, Jul-06, May-09 | MG, PN, JS, AS | KJ743015, KJ743017, KJ743020, KJ743025, KJ743044, KJ743046, KJ743048, KJ743102-17 | 6(7), 19(11), 39, 40, 41, 42, 43 |
|  |  |  |  |  |  |  |  |  |

PN Paul Nichols, MG Martin Genner, GFT George Turner, JS Jennifer Swanstrom, AS Alan Smith

*indicates samples also screened for microsatellite DNA allele frequency variation (Table S

**Table S2. Sampling localities and numbers of individuals genotyped at microsatellite DNA loci.**

| Sampling location | Latitude | Longitude | n | Sampling Date | Collector |
| --- | --- | --- | --- | --- | --- |
| Salima | 13° 46' 9'' S | 34° 26' 8'' E | 33 | Apr-04 | MG, GFT |
| Ruvuma* (Mangochi Hills, Kwiputi) | 14° 22' 27'' S | 35° 32' 55'' E | 30 | May-09 | PN |
| Liwonde (LMC) | 15° 3' 13'' S | 35° 13' 10'' E | 28 | Jul-05 | PN |
| Liwonde (EC) | 15° 3' 13'' S | 35° 13' 10'' E | 46 | Jul-05 | PN |
| Liuli Dam | 16° 2' 16'' S | 34° 50' 27'' E | 32 | Jul-05 | PN |

*also referred to as Rovuma elsewhere

**Table S3: Microsatellite-based estimates of genetic differentiation between populations of *A. calliptera*.** Below the diagonal pairwise *F*_ST_, above the diagonal *P* statistics.

| Sampling location | Salima | Ruvuma | Liwonde (LMC) | Liwonde (EC) | Liuli Dam |
| --- | --- | --- | --- | --- | --- |
| Salima |  | < 0.001 | < 0.001 | < 0.001 | < 0.001 |
| Ruvuma | 0.2393 |  | < 0.001 | < 0.001 | < 0.001 |
| Liwonde (LMC) | 0.0963 | 0.1252 |  | 0.5354 | < 0.001 |
| Liwonde (EC) | 0.0834 | 0.1368 | 0.0001 |  | < 0.001 |
| Liuli Dam | 0.1346 | 0.1899 | 0.0556 | 0.0520 |  |

**Table S4. Spawning decisions.** Number of eggs sired by individual males in each of 4 replicate experiments. Blue numbers are the number of eggs South-Eastern catchment females spawned with South-Eastern catchment males and red numbers are the number of eggs Lake Malawi catchment females spawned with Lake Malawi catchment males. Numbers in shaded areas represent spawning decisions which lead to hybridization between the two lineages.

|  |  | Ruvuma | | | Salima | | |
| --- | --- | --- | --- | --- | --- | --- | --- |
|  | ♀ | ♂ | ♂ | ♂ | ♂ | ♂ | ♂ |
| Rep1 |  | 1 | 2 | 3 | 1 | 2 | 3 |
| South Eastern catchment (Ruvuma) | 1 |  |  |  | 6 |  |  |
|  | 2 | **7** |  |  |  |  |  |
|  | 3 | **7** |  |  |  |  |  |
|  | 4 | **7** |  |  |  |  |  |
|  | 5 |  |  |  | 7 |  |  |
|  | 6 |  |  | **8** |  |  |  |
| Lake Malawi catchment (Salima) | 1 |  |  |  |  |  | **7** |
|  | 2 |  |  |  | **8** |  |  |
|  | 3 |  |  |  | **6** |  |  |
|  | 4 |  |  |  |  |  |  |
|  | 5 |  |  |  |  |  | **8** |
|  | 6 |  |  |  | **8** |  |  |
| Rep2 |  | 4 | 5 | 6 | 4 | 5 | 6 |
| South Eastern catchment (Ruvuma) | 7 |  | **8** |  |  |  |  |
|  | 8 |  |  | **7** |  |  |  |
|  | 9 |  |  | **8** |  |  |  |
|  | 10 |  |  | **8** |  |  |  |
|  | 11 |  |  |  |  |  | 6 |
|  | 12 |  |  |  |  |  |  |
| Lake Malawi catchment (Salima) | 7 |  |  |  |  |  | **8** |
|  | 8 |  | 4 |  |  |  |  |
|  | 9 |  |  |  |  |  | **8** |
|  | 10 |  |  |  |  | **8** |  |
|  | 11 |  |  |  |  |  | **6** |
|  | 12 |  |  |  |  |  |  |
| Rep3 |  | 7 | 8 | 9 | 7 | 8 | 9 |
| South Eastern catchment (Ruvuma) | 13 |  |  |  |  |  |  |
|  | 14 |  |  |  |  |  |  |
|  | 15 |  |  | **8** |  |  |  |
|  | 16 |  |  | **4** |  | 7 |  |
|  | 17 | **6** |  |  |  |  |  |
|  | 18 |  |  |  |  |  |  |
| Lake Malawi catchment (Salima) | 13 |  |  |  |  |  | **8** |
|  | 14 |  |  |  |  |  | **7** |
|  | 15 |  |  |  |  |  | **4** |
|  | 16 |  |  | 8 |  |  |  |
|  | 17 |  |  |  |  |  |  |
|  | 18 |  |  |  |  |  |  |
| Rep4 |  | 10 | 11 | 12 | 10 | 11 | 12 |
| South Eastern catchment (Ruvuma) | 19 |  |  |  |  |  |  |
|  | 20 |  |  | **8** |  |  |  |
|  | 21 |  |  |  |  |  |  |
|  | 22 |  |  |  |  |  |  |
|  | 23 |  | **7** |  |  |  |  |
|  | 24 |  |  |  |  |  |  |
| Lake Malawi catchment (Salima) | 19 |  |  |  |  |  | **7** |
|  | 20 |  |  |  |  | **8** |  |
|  | 21 |  |  |  | **8** |  |  |
|  | 22 | 8 |  |  |  |  |  |
|  | 23 |  |  |  |  | **8** |  |
|  | 24 |  |  |  |  | **8** |  |

**Table S5. Experimental crosses to reconstruct hybridization.**

| **F1 Generation** |  | Maternal lineage | | | |
| --- | --- | --- | --- | --- | --- |
| Paternal lineage |  | *A. calliptera* 'L. Malawi' LMC | | *A. calliptera* 'Ruvuma' SEC | |
|  |  | - L | | - R | |
| *A. calliptera* 'L. Malawi' | -L | LL | | LR | |
| *A. calliptera* 'Ruvuma' | -R | RL | | RR | |
| **F2 Generation** |  | LL | LR | LR | RR |
|  | LL | LLLL | LLLR | LLLR | LLRR |
|  | LR | LRLL | LRLR | LRLR | LRRR |
|  | RL | RLLL | RLLR | RLLR | RLRR |
|  | RR | RRLL | RRLR | RRLR | RRRR |

**Table S6. Number of individuals analysed for morphology in each brood**

| Cross Type | Cross details* | Brood ID | Individuals |
| --- | --- | --- | --- |
| LMC | LLLL | B5 | 9 |
|  |  | B9 | 19 |
|  | *Total Broods* | *2* | **28** |
| LMC x F1 | LLLR | B21 | 19 |
|  | LLLR | B28 | 16 |
|  | LLRL | B14 | 19 |
|  | LLRL | B19 | 9 |
|  | LRLL | B13 | 22 |
|  | LRLL | B16 | 8 |
|  | RLLL | B8 | 25 |
|  | RLLL | B24 | 10 |
|  | *Total Broods* | *8* | **128** |
| SEC | RRRR | B1 | 12 |
|  |  | B20 | 10 |
|  | *Total Broods* | *2* | **22** |
| F1 | LLRR | B12 | 20 |
|  | RRLL | B2 | 19 |
|  | RRLL | B18 | 19 |
|  | *Total Broods* | *3* | **58** |
| F2 | LRLR | B6 | 20 |
|  | LRLR | B17 | 29 |
|  | LRRL | B11 | 19 |
|  | RLLR | B7 | 20 |
|  | RLLR | B31 | 20 |
|  | RLRL | B3 | 15 |
|  | RLRL | B29 | 13 |
|  | *Total Broods* | *7* | **136** |
| SEC x F1 | LRRR | B22 | 16 |
|  | RLRR | B15 | 20 |
|  | RLRR | B23 | 21 |
|  | RRLR | B27 | 11 |
|  | RRRL | B4 | 10 |
|  | RRRL | B10 | 8 |
|  | RRRL | B30 | 18 |
|  | *Total Broods* | *7* | **104** |

*L=LMC, Lake Malawi Catchment fish from Salima stock.

*R=SEC, South-eastern Catchment fish from Ruvuma stock.

**Table S7. Associations between relative warp (shape) axes and body size (centroid size) of laboratory reared experimental fish, corrected prior to analyses presented in Table 1**

| RW axis | r^2^ | F | p |
| --- | --- | --- | --- |
| 1 | 0.085 | 43.759 | < 0.001 |
| 2 | 0.066 | 33.373 | < 0.001 |
| 3 | 0.010 | 4.707 | 0.031 |
| 4 | 0.044 | 21.570 | < 0.001 |
| 5 | 0.032 | 15.446 | < 0.001 |
| 6 | 0.095 | 49.813 | < 0.001 |

**Table S8- Wild samples analysed for morphology.** Geometric morphometrics were conducted on these 52 wild individuals following methods described in the main text.

| Location | Latitude | Longitude | n | Sampling Date(s) | Collector(s) |
| --- | --- | --- | --- | --- | --- |
| Salima | 13° 46' 9'' S | 34° 26' 8'' E | 15 | Jan-11 | MG, PP |
| Ruvuma | 14° 22' 27'' S | 35° 32' 55'' E | 17 | Oct-04, Sept-12 | MG, PN, PP, JS, HS |
| Liwonde | 15° 3' 13'' S | 35° 13' 10'' E | 20 | Jan-11, Sept-12 | MG, PN, JS, HS |

**Table S9 . Associations between relative warp axes and body size (centroid size), corrected prior to analyses presented in Table 1**

| RW axis | r^2^ | F | p |
| --- | --- | --- | --- |
|  |  |  |  |
| 1 | 0.132 | 7.639 | 0.007 |
| 2 | 0.010 | 0.511 | 0.477 |
| 3 | 0.099 | 5.493 | 0.023 |
| 4 | 0.108 | 6.037 | 0.017 |
| 5 | 0.058 | 3.097 | 0.084 |
| 6 | 0.034 | 1.746 | 0.192 |
|  |  |  |  |

**Table S10: Extent of novel phenotypic space observed in Liwonde relative along six primary axes of body-size corrected morphospace**.

| Relative Warp  Axis | % novel space in Liwonde relative to Ruvuma and Salima combined |
| --- | --- |
|  |  |
| RW1 | 19.70 |
| RW2 | 4.07 |
| RW3 | 79.94 |
| RW4 | 11.79 |
| RW5 | 0 |
| RW6 | 0 |
|  |  |

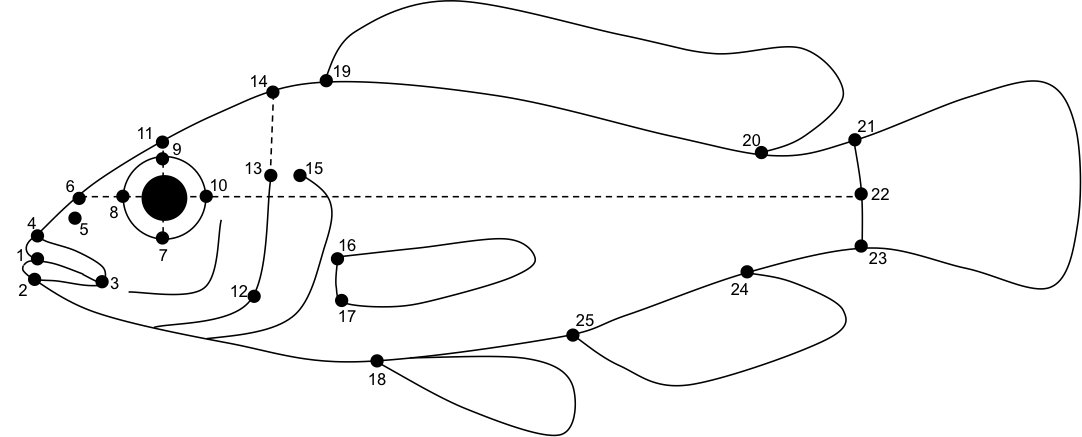


**Figure S2.** Points used for landmark based image analysis. Numbered points are as follows:1) tip of the anterior-most tooth on the premaxilla 2) anterior tip of dentary 3) anterior reach of the premaxillary groove 4) anterior tip of maxilla, 5) nostril 6) intersection of the horizontal axis of the eye with the anterior limit to the body 7) ventral intersection of the vertical axis of the eye, 8) anterior intersection of the horizontal axis of the eye, 9) dorsal intersection of the vertical axis of the eye, 10) posterior intersection of the horizontal axis of the eye, 11) intersection of the vertical axis of the eye with the dorsal limit to the body, 12) lower margin of preopercule 13) upper margin of preopercule 14) junction of preopercule with dorsal limit to the body, 15) top of operculum, 16) ventral insertion of pectoral fin, 17) dorsal insertion of pectoral fin,18) insertion of pelvic fin19) anterior insertion of dorsal fin, 20) posterior insertion of dorsal fin, 21) dorsal junction of caudal fin and caudal peduncle, 22) centre of junction of caudal fin with caudal peduncle, 23) ventral junction of caudal fin and caudal peduncle, 24) posterior insertion of anal fin, 25) anterior insertion of anal fin.

**Figure S3.** Morphological variation in wild caught specimens from Liwonde, Ruvuma and Salima. See Table S8 for sampling details.
